# Human cord blood-derived mononuclear cell transplantation for viral encephalitis-associated cognitive impairment: a case report

**DOI:** 10.1186/1752-1947-7-181

**Published:** 2013-07-08

**Authors:** Wan-Zhang Yang, Guo-Jian Shu, Yun Zhang, Fang Wu, Bi-Yu Ye, Xiang Hu

**Affiliations:** 1 Department of Rehabilitation Medicine, Nanshan Affiliated Hospital of Guangdong Medical College, Shenzhen, China; 2 Shenzhen Beike Cell Engineering Research Institute, 2/F, Yuanxing Technology Building, #1 Songpingshan Street, North Area, Hi-Tech Industrial Park, Shenzhen 518057, China

## Abstract

**Introduction:**

Herpes simplex virus is the most common cause of sporadic viral encephalitis. Cognitive impairments persist in most patients who survive herpes simplex virus-caused encephalitis after undergoing currently available treatments. This is the first report on the development of human cord blood-derived mononuclear cell transplantation as a new treatment intervention to improve the prognosis of sequelae of viral encephalitis.

**Case presentation:**

An 11-year-old Han Chinese boy developed sequelae of viral encephalitis with cognitive, mental and motor impairments in the 8 months following routine treatments. Since receiving allogeneic cord blood-derived mononuclear cell transplantation combined with comprehensive rehabilitation therapies 7 years ago, the patient’s health has significantly improved and remained stable.

**Conclusions:**

Human cord blood-derived mononuclear cell transplantation may be a potential therapeutic strategy for treating the neuropsychiatric and neurobehavioral sequelae of viral encephalitis.

## Introduction

Viral encephalitis is a diffuse or focal inflammatory process of the brain parenchyma caused by the infection of a variety of viruses. It commonly presents with fever, headache, seizures, disturbance of consciousness, and dysfunction of movement, intelligence, and language induced by focal central nervous system damage in the acute stage [1]. Worldwide data indicate an annual incidence of acute encephalitis ranging from 3.5 to 7.4 out of 100,000, rising to 16 out of 100,000 in children [2]. There are numerous potential causes of viral encephalitis and the causes of 30% to 50% of cases may not be identified even after extensive investigation [3]. Therefore, the diagnosis of viral encephalitis can be defined if the patient has the appropriate clinical presentation and surrogate markers of brain inflammation, such as inflammatory cells in the cerebrospinal fluid (CSF) or changes to brain imaging suggestive of inflammation [2,4]. Herpes simplex virus (HSV) is the most common cause of sporadic viral encephalitis [2]. Most patients with HSV-caused encephalitis (HSE) present with mental and cognitive impairment and a long duration of the aforementioned symptoms [5]. Current treatment methods for viral encephalitis include the use of an antiviral drug with good CSF penetration when the infection is caused by a treatable virus, immunomodulatory treatment when indicated, and prevention and control of the complications of the disease [1,4]. However, it has been reported that cognitive and other neuropsychiatric features persist in as many as 80% or more of patients who have survived HSE [5].

One patient diagnosed with the sequelae of viral encephalitis was admitted into the Nanshan Affiliated Hospital of Guangdong Medical College (Shenzhen, China) to seek stem cell transplantation due to cognitive, language and mental impairments in the 8 months following routine treatments for viral encephalitis. After receiving allogeneic cord blood-derived mononuclear cell (CBMC) transplantation combined with comprehensive rehabilitation therapies, the patient obtained significant improvements and maintained a stable condition for the following 5 years.

## Case presentation

Eight years ago, an 11-year-old Han Chinese boy was admitted to a local hospital with acute onset of "high fever and coma". A physical examination showed high body temperature, unconsciousness and recurrent seizures. The diagnosis of “viral encephalitis complicated with secondary epilepsy” was defined according to the symptoms and examination results of CSF, electroencephalography (EEG) and brain magnetic resonance imaging (MRI). The patient received approximately 2 months of anti-virus, seizure control, and supportive and symptomatic treatments while in the hospital. He was unable to speak, lacked normal voluntary control of excretory functions, and displayed emotional irritability upon discharge. An EEG test before discharge revealed diffuse 2~3Hz δ activity with middle to high amplitude waves within the brain (no remarkable change to the previous test result). The patient’s condition was not substantially changed by other treatment interventions after discharge, including rehabilitation therapies.

Seven years ago, the patient was admitted to the Nanshan Affiliated Hospital of Guangdong Medical College for stem cell treatment due to “sequelae of viral encephalitis complicated with secondary epilepsy and cognitive impairment”. A physical examination showed: (1) Consciousness, but with severe cognitive impairment on memory, thinking, understanding, and calculation; (2) Mental disorders, presenting with panic, irritability, shouting and crying; (3) Transcortical aphasia with alexia, and agraphia; (4) Muscular tone of his four limbs was grade 1^-^ (Ashworth scale) with apraxia and the patient was incapable of walking up or down stairs; (5) Evaluation of “Activities of Daily Living” (ADL) revealing complete dependence of feeding, urination and defecation with a score of “0” on the Barthel Index (BI); (6) “0” score on “Functional Independence Measure” (FIM); (7) No cooperation for “Mini-Mental State Examination” (MMSE); (8) “0” score on “Wechsler Intelligence Scale for Children” (WISC) (Table 1). The blood test results were within normal range and the brain MRI showed white matter lesions on the bilateral hemispheres. The patient was not taking any medication on admission.

**Table 1:** Evaluation of treatment efficacy: four scales pre- and post-treatment

**Scale**	**ADL-BI**	**FIM**	**MMSE**	**WISC**
Pre-treatment	0	0	No cooperation	0
Post-treatment	100	123	29	73

Abbreviations: *ADL* Activities of Daily Living, *BI* Barthel Index, *FIM* Functional Independence Measure, *MMSE* Mini-Mental State Examination, *WISC* Wechsler Intelligence Scale for Children.

The patient received 6 allogeneic CBMC transplantations in combination with rehabilitation therapies. The treatment protocol and patient consent were approved by the local Institutional Review Board of the Nanshan Affiliated Hospital of Guangdong Medical College under the auspices of the National Ministry of Health. The treatment procedure was clearly explained to the patient’s family and informed consent was obtained before the initiation of each cell transplantation. The CBMCs were provided by Shenzhen Beike Biotechnology Co. Ltd. after human umbilical cord blood collection and mononuclear cell extraction, cultivation and harvesting [6]. To ensure the quality of CBMCs, a number of parameters were tested and confirmed before use, including hepatitis B virus, hepatitis C virus, human immunodeficiency virus, alanine aminotransferase, syphilis and endotoxin level. Approximately 1~3×10^7^ CBMCs (containing 1.0% to 2.0% CD34^+^ cells, cell viability ≥95%) were transfused per injection at 1-week intervals. The patient received four cell infusions through intrathecal injection and two cell infusions by intravenous injection. Rehabilitation therapies, including scalp acupuncture [7] and speech and cognitive training with the CE-1275 vocaSTIM®-Master instrument (Physiomed GMDH, Germany), were applied over three treatment courses with the duration of each course lasting 20 days (once per day). No medication was commenced during the treatment and on discharge from the hospital.

The patient displayed remarkable improvements after the 6 stem cell transplantations and 3 months of comprehensive rehabilitation therapies. He was slightly malnourished when admitted and his appetite and eating habits improved after stem cell treatment. Additional improvements were as follows: (1) Cognition – completely normal function of calculation, long-term memory, orientation and thinking, except for slightly inadequate short-term memory; (2) Language – completely recovered speaking, reading, writing and listening ability; (3) Movement – completely recovered motor function; (4) Normal mental condition without irritability or panic; (5) ADL – completely independent daily life with “100” BI score; (6) FIM score of 123, largely independent; (7) MMSE score of 29, effectively normal; (8) WISC score of 73, normal intelligence (Table 1); (9) Video-EEG (June 2006) test revealed significant improvements compared with previous test results. A brain MRI scan was not performed post-treatment.

The patient’s condition has not only remained stable since discharge but also gradually improved to the point that he was able to return to school to continue his studies. He was able to catch up with the teaching materials and his exam scores for major subjects were a B^+^. He successfully enrolled in high school 5 years later. Twelve tumor indicators, including carbohydrate antigen (CA) 19–9, CA242, CA-125, CA 15-3, neuron-specific enolase, carcinoembryonic antigen, alpha-fetoprotein, total prostate-specific antigen (PSA), free PSA, ferritin, β-human chorionic gonadotropin and growth hormone, were within the normal range when tested at 2 years post-treatment (Table 2). In the 5-year follow-up, the patient showed a height of 175cm, normal physiological indexes, cognitive and language capacity, and normal test results of tumor indicators (Table 2). These therapeutic effects have been fully affirmed by clinicians from other non-related hospitals when performing their own independent checks on the patient.

**Table 2:** Twelve tumor indicators at 2 and 5 years post-treatment

**Item**	**Reference range**	**2-year post-treatment**	**5-year post-treatment**
CA 19-9 (u/mL)	<35.0	2.57	2.68
CA 242 (ng/mL)	<20.0	1.70	2.40
CA125 (u/mL)	<35.0	8.82	7.05
CA15-3 (u/mL)	<35.0	6.42	9.48
NSE (ng/mL)	<13.0	1.70	2.40
CEA (ng/mL)	<5.0	3.33	4.90
AFP (ng/mL)	<20.0	2.16	1.71
PSA (ng/mL)	<5.0	0.41	0.22
free PSA (ng/mL)	<1.0	0.16	0.09
Ferritin (ng/mL)	<322.0	77.59	69.67
β-hCG (ng/mL)	<3.0	0.24	0.29
GH (ng/mL)	<7.50	0.17	0.10

Abbreviations: *AFP* alpha-fetoprotein, *β-hCG* β-human chorionic gonadotropin, *CA* carbohydrate antigen, *CEA* carcinoembryonic antigen, *GH* growth hormone, *NSE* neuron-specific enolase, *PSA* total prostate-specific antigen.

## Discussion

This patient was diagnosed with viral encephalitis. The results of both the EEG and brain MRI indicated diffuse lesions in the whole brain and the natural cure of these lesions was unsubstantial. The patient presented with severe disturbances of advanced brain functions on language, cognition, and thinking in the 8 months following routine treatments, leading to a life of complete dependency. After receiving 6 allogeneic CBMC transplantations combined with comprehensive rehabilitation therapies, the patient regained normal function of his language, thinking, understanding, long-term memory, and cognition. In the 5 years of follow ups since the stem cell treatment, the patient’s physiological indicators were shown to be in the normal ranges and his capacity for thinking, language, and mental awareness was restored and maintained at a level similar to that of before the onset of the disease. These preliminary clinical data suggest that stem cell transplantation may be a new treatment option for the severe sequelae of viral encephalitis.

We have previously reported on the safety and freedom from immunologically mediated adverse effects of allogeneic CBMC therapy in the absence of immune suppression and myeloablation [6]. This patient’s blood test results of tumor indicators and his physical condition demonstrated no evidence of tumor formation during the 5 years of follow ups. This case further demonstrates the long-term safety of using non-matched, allogeneic cord blood cells to treat non-hematopoietic conditions. It also indicates that CBMC transplantation may be a safe and effective therapy intervention for the sequelae of viral encephalitis.

Abundant animal research has demonstrated that administered cord blood stem cells can enter the brain, survive, and migrate to damaged areas to improve functional recovery for various neurological diseases [8-10]. A previous case series study of 30 patients with hereditary ataxia indicated that treatment using both intravenous and intrathecal infusion of CBMCs combined with rehabilitation therapy improved ataxia along with functionality and quality of life [11]. Mechanism studies suggest multi-potent cells in the heterogeneous CBMC population may not only differentiate into neurons and astrocytes to act as a cell replacement source, but also produce antioxidants, several neurotrophic and angiogenic factors and modulate immune and inflammatory reaction [12,13]. CBMC graft-mediated brain repair involves neurotrophic effects resulting from the release of various growth factors that afford cell survival, angiogenesis, and anti-inflammation to rescue the injured nerves and support the regeneration of neurons and glial cells [14]. These multiple restorative and protective effects from CBMC transplantation may be interdependent and act in harmony to exert therapeutic benefits in this case. However, the exact mechanisms of action need to be further explored in the future with basic and clinical studies.

Modern rehabilitation theory believes rehabilitation therapy can aid regeneration of damaged neurons and functional plasticity of the central nervous system after brain injury [15-17]. Furthermore, rehabilitation therapy may exert a synergistic action on grafted cells to promote functional recovery [11,18,19]. Recent studies have demonstrated that rehabilitation therapy can activate the survival and differentiation of grafted cells, initiate synthesis and secretion of endogenous neurotrophic factors in the ambient tissues at the lesion sites, and down-regulate expression of glial fibrillary acidic protein and chondroitin sulfate proteoglycan protein to prevent axonal degeneration and improve axonal regeneration [20,21]. Therefore, we adopted comprehensive rehabilitation therapies to combine with the CBMC transplantation to assist in the promotion of the patient’s functional recovery.

## Conclusions

Viral encephalitis is still considered a major medical problem. Severe neurological sequelae are common even though correct diagnosis and adequate antiviral therapy have been performed. There is no valid treatment intervention able to completely cure the cognitive dysfunction patients suffer due to viral encephalitis. The development of new treatment intervention to improve the prognosis of the sequelae of viral encephalitis is needed. This case report has indicated that CBMC transplantation combined with rehabilitation therapy might be a potential therapeutic strategy for the neuropsychiatric and neurobehavioral sequelae of viral encephalitis. Future clinical studies with more samples are needed to further validate safety and treatment efficacy.

## Consent

Written informed consent was obtained from the patient’s legal guardian for publication of this case report. A copy of the written consent is available for review by the Editor-in-Chief of this journal.

## Abbreviations

ADL: Activities of Daily Living; BI: Barthel index; CA: Carbohydrate antigen; CBMC: Cord blood-derived mononuclear cell; CSF: Cerebrospinal fluid; EEG: Electroencephalography; FIM: Functional Independence Measure; HSE: HSV-caused encephalitis; HSV: Herpes simplex virus; MMSE: Mini-Mental State Examination; MRI: Magnetic resonance imaging; PSA: Prostate-specific antigen; WISC: Wechsler Intelligence Scale for Children.

## Competing interests

XH is a shareholder of Shenzhen Beike Cell Engineering Research Institute. YZ is an employee of Shenzhen Beike Cell Engineering Research Institute. No other authors declare any competing interests.

## Authors’ contributions

WY, GS, FW, and BY carried out the clinical treatment and follow-up and collected data. YZ and XH analyzed and interpreted data and drafted the manuscript. All authors read and approved the final manuscript.
